# Evaluation of Contamination and Ecological Risk of Heavy Metals Associated with Cement Production in Ewekoro, Southwest Nigeria

**DOI:** 10.5696/2156-9614-10.25.200306

**Published:** 2020-02-28

**Authors:** Temitope Ayodeji Laniyan, Adeniyi JohnPaul Adewumi

**Affiliations:** 1 Department of Environmental Health Sciences, Faculty of Public Health, College of Medicine, University of Ibadan, Ibadan, Nigeria; 2 Department of Geological Sciences, Achievers University, Owo, Ondo State, Nigeria

**Keywords:** cement production, contaminated, ecological, heavy metals mobilized, soil

## Abstract

**Background.:**

Exposure to heavy metals emanating from cement production and other anthropogenic activities can pose ecological risks.

**Objectives.:**

A detailed investigation was carried out to assess the contamination and ecological risk of heavy metals associated with dust released during cement production.

**Methods.:**

Sixty samples, including 30 soils and 30 plants, were collected around Lafarge Cement Production Company. Control samples of soil and plants were collected in areas where human activities are limited. Samples were dried, sieved (for soil; 65 μm), packaged and analyzed using inductively coupled plasma mass spectrometry at Acme Laboratory in Canada.

**Results.:**

The average concentration of heavy metals in soils of the area are: copper (Cu): 41.63 mg/kg; lead (Pb): 35.43 mg/kg; zinc (Zn): 213.64 mg/kg; chromium (Cr): 35.60 mg/kg; cobalt (Co): 3.84 mg/kg and nickel (Ni): 5.13 mg/kg. Concentrations of Cr in soils were above the recommended standards, while other metals were below recommended limits. The average concentrations of heavy metals in plants were: Cu: 26.32 mg/kg; Pb: 15.46 mg/kg; Zn: 213.94 mg/kg; Cr: 30.62 mg/kg; Co: 0.45 mg/kg and Ni: 3.77 mg/kg. Levels of heavy metals in plants were all above international limits. Geo-accumulation of metals in soils ranged between −0.15 and 6.32, while the contamination factor ranged between 0.53 and 119.59. Ecological risk index of heavy metals in soils ranged between 49.71 and 749.

**Discussion.:**

All metals in soils of the study area except for Cr were below the allowable limits, while the levels of metals in plants were above the permissible limits. Levels of heavy metals reported in this study were higher than those from similar cement production areas. Soils around the Ewekoro cement production area were low to extremely contaminated by toxic metals. Cement production, processing, transportation in conjunction with the abandoned railway track in the area greatly contribute to the high degree of contamination observed in the area. Metal transfers from soil to plant are a common phenomenon. The metals pose low to considerable ecological risk.

**Conclusions.:**

Anthropogenic sources, especially cement processing activities, release heavy metals which leads to progressive pollution of the environment and poses high ecological risk.

**Competing Interests.:**

The authors declare no competing financial interests

## Introduction

Emission of toxic metals from industries is a source of environmental degradation, and can affect human heath.^1–10^ Cement production emits dust particles of various sizes, volatile substances and dangerous metals, harming the environment.^11^,^12^ Cement dust reduces crop yield (through stomata clogging), gaseous exchange, rate of transpiration and inhibits intercellular processes, and also affects surrounding ecosystems.^13–17^ The main materials used in the production of cement are limestone, shells, and chalk or marl combined with shale, clay, slate, blast furnace slag, silica sand, and iron ore.^18^ Metals and compounds, such as lead, zinc, and sulfuric acid, originate from cement manufacturing plants.^19^

In developing countries, like Nigeria, soil quality plays a crucial role in food production, as metals emitted from industries can bioaccumulate in plants from soil.^20–23^ Ingestion of these plants can lead to health problems and eventually mortality.^20–26^ Cement dust has a high percentage of calcium silicate which is harmful to human health when ingested.^27^,^28^ The alkaline compound has the ability to transform to C-S-H bond when it reacts with oxygen in the soil, this consequently makes the presence of calcium silicate a major problem when ingested or even with dermal contact.^29^

Cement dust also contains hexavalent chromium, a compound that is highly toxic in nature, and has major health impacts when bioaccumulation occurs up the food chain.^35–37^

Little or no study has been carried out to assess the extent of contamination of heavy metals in soils and plants or evaluate the health and ecological risks associated with cement production in the study area.^38–40^ The research was therefore carried out to assess the impact of heavy metals found in cement dust on the soils and plants within and around Ewekoro Portland cement factory and also evaluates the health impacts.

## Methods

**Figure 1:**
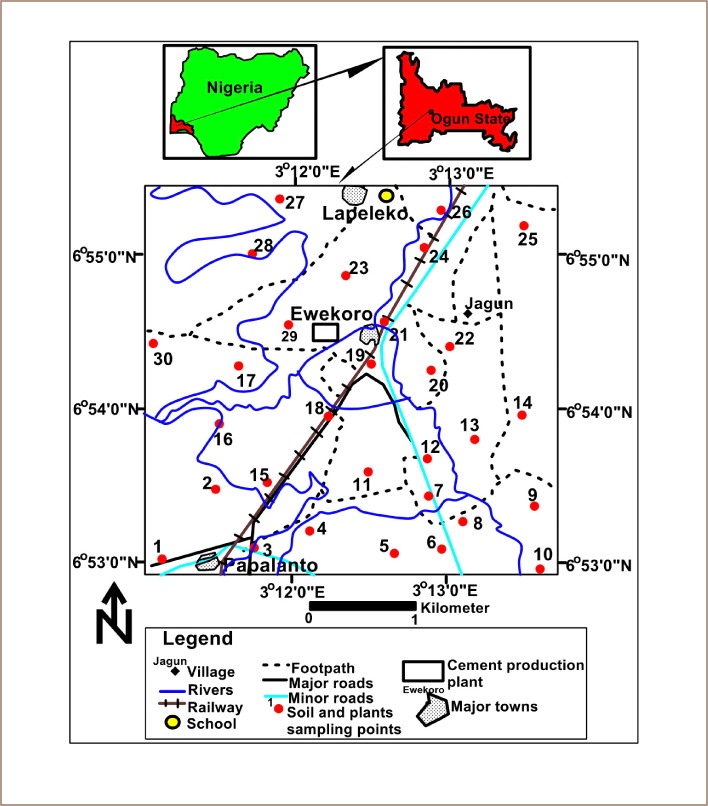
Map of Study Area

Ewekoro is located within latitude N 6°53′00″-N 6°55′00″ and longitude E 3°12′00″-E 3°13′00″ in southwestern Nigeria *(Figure 1).* Ewekoro is found along the Sango-Ifo-Abeokuta Expressway of Ogun State, bordering Papalanto in the west and Abeokuta in the east. The town is 54 km from Lagos and 24 km from Abeokuta. Ewekoro is easily accessible and drained mainly by the River Ewekoro, which is seasonal in nature and has many tributaries.^41^ The topography of Ewekoro is an immeasurable low land. The area experiences high levels of humidity and shrubbery is primarily located on tree plantations. The climate is significantly marked by two alternating wet and dry seasons. The average temperature in the area is 27.1°C with the highest and lowest temperatures recorded in March and August, respectively. The mean annual rainfall in the area is 1305 mm with the highest rainfall observed in June and the lowest rainfall in January.^42^ According to the United States Department of Agriculture classification scheme, the soils of the area are ferric, quartz and highly weathered clay minerals.^43^,^44^ Human activities in the area include mining, quarrying, farming and cement production, and has a population of 55,156.^45^,^46^ The area is found within the Dahomey basin, which is one of the major sedimentary basins in Nigeria. Ewekoro is a type locality for limestone deposits in the country and soil type reflects the presence of clay minerals.^47^,^48^

**Abbreviations**
*CD*: Contamination degree
*CF*: Contamination factor
*ER*: Ecological risk
*ERI*: Ecological risk index
*Igeo*: Geo-accumulation index
*PLI*: Pollution load index
*RI*: Risk index

### Sampling

Thirty (30) topsoil samples weighing 1 kg each were collected at a depth of 20 cm in March 2015 using a hand auger. After each sample was collected, all instruments used for sample collection were washed using distilled water and dried before use at the next sampling point.^49^ This method was repeated for of all of the collected samples. Three control samples were collected in areas with no observed anthropogenic activities. Likewise, 30 healthy plant samples, commonly consumed by locals were collected from four species of plants: Celosia argentea (soko), Corchorus olitorius (ewedu), Colocasiaesculenta (cocoyam), Musa sp.(banana) and Saccharum officinarum (sugar cane). The foliage was picked into a container and tagged.^50^,^51^ Two (2) control samples of soils and plants were also collected in areas with no observed anthropogenic activities.

### Chemical analysis

In the laboratory, all the soil and plant samples were dried at room temperature. The sods were pulverized and sieved using an impact electric sieve shaker. After sieving, clay-sized (63 μm) sediments were collected and packed into small zip-lock bags. The grains were divided into roots, stems, and leaves and pulverized to fineness (<0.002 mm) using a china clay mortar and pestle. Soil and plant samples were then digested before analysis.

One (1) gram of soil was weighed from each pulverized sample and dissolved with 15 ml nitric acid, 20.0 ml perchloric acid and 15.0 ml hydrofluoric acid, and heated for three hours and thereafter measured into a 100 ml flask with distilled water.^51^ Plant samples were thoroughly washed in deionized water because they are more prone to battering of sediments, heated at 105°C for 5 minutes, cooled at 70°C for 48–72 hours to a stable mass, then turned into a powdery form in a 100 μm blender so they could be easily dissolved.^52–54^ A homogenized measured mass of 0.25 g was put in a 100-ml dry Pyrex digestion tube and digested with 5 ml of concentrated nitric acid was measured with it for the metals analysis. Digested samples were diluted with ultrapure water using a 1:50 dilution factor. Digestates were sent for analysis at Acme Laboratory Canada. Metals analyzed in the samples included copper (Cu), lead (Pb), zinc (Zn), chromium (Cr), cobalt (Co) and nickel (Ni). Statistical analyses were carried out using the Statistical Package for the Social Sciences (SPSS) software program version 21. It was used to calculate mean, minimum, maximum, standard deviation and bivariate correlation.

### Contamination and risk assessment

The equations described below were used to evaluate the contamination, ecological and health risks of heavy metals in samples. Heavy metal contamination in soils was calculated using the geo-accumulation index (Igeo), contamination factor (CF), contamination degree (CD) and pollution load index (PLI).

### Geo-accumulation index

The Igeo was used to assess contamination of a specific metal in soils by evaluating metal enrichment above baseline or background values. Geo-accumulation index was calculated according to Equation 1.^55^

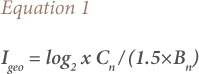
where, C_n_ is the metal concentration in the sample; B_n_ is the concentration of metal in the background sample, and the constant 1.5 is introduced to minimize the effect of possible variations in the background values which may be attributed to lithologic variations in the samples. The following interpretation for the Igeo was given by Loska*et al*.: Igeo<0 = practically unpolluted, 0<Igeo<1 = unpolluted to moderated polluted, 1<Igeo<2 = moderately polluted, 2<Igeo<3 = moderately to strongly polluted, 3<Igeo<4=strongly polluted, 4<Igeo<5= strongly to extremely polluted and Igeo>5 = extremely polluted.^56^

### Contamination factor

The assessment of soil contamination was also carried out using the CF in Equation 2. The CF is the single element index, and all four classes are recognized.^57^

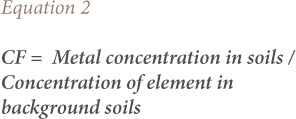
The CF can be classified as follows: CF<1: low contamination; 1<CF<3: moderate contamination; 3<CF<6: considerable contamination; CF≥6: very high contamination.

### Contamination degree

The sum of contamination factors for all examined elements represents the CF of the environment.^58^ The CD is aimed at providing a measure of the degree of overall contamination in surface layers in a particular sampling site. The formula for calculating the CD is shown in Equation 3.

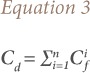
where, C_d_ is the contamination degree and C_f_ is the contamination factor. A CD<6 indicates a low degree of contamination, while 6<CD<12 implies a moderate degree of contamination. In addition, 12<CD<24 indicates a considerable degree of contamination, while CD> 24 reflects a high degree of contamination.

### Pollution load index

The PLI is defined as the ratio of element concentration in the study to the background content of the abundance of chemical elements in the continental crust and is used to assess environment quality.^59^,^60^ The PLI for the soil samples was determined by the equation below, as proposed by Tomilson *et al.* and used by Anjos *et al.*^58^,^59^

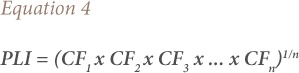
According to Chen *et al.*, the PLI of each metal is classified as either low (PI≤1), middle (1<PI≤3) or high (PI>3).^60^

### Contamination load index

Equation 5 was used to determine the rate of contamination of specific metals in the grain/plant.

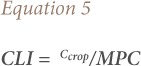
Where, CLI is the contamination load index; C_crop_ is the concentration of metal in a plant; MPC is the maximum permitted concentration of metals in crops, and a contamination load index >1 indicates contamination of grains by metals.^61^

### Bioaccumulation factor

Bioaccumulation factor is defined as the ratio of metal concentration in plant to that in the soil.^62^ It is expressed using Equation 6.

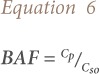
Where, BAF is the bioaccumulation factor, and C_p_ and C_so_ are the metal concentration in aerial parts of the plant (mg/kg) and in soil (mg/kg), respectively. When the bioaccumulation factor>1 there is mobility of metal from soil to plant.

### Ecological risk assessment for metals in soils

Ecological risks of metals were evaluated using the ecological risk index (ERI) *(Equation 7)* as presented by Mamut *et al.*^63^

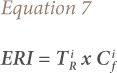
Where, ERI is the potential ecological risk of a single element;T_R_ is the toxic-response factor; and C_F_ is the pollution of a single element factor, which is also the contamination factor. The toxic-response factors for some metals used in the study were Zn = 1, Cr = 2, Cu = 5, Pb = 5, cadmium (Cd) = 30, Ni = 5. The results from Equation 7 help to produce the risk index (RI), which is the summation of the ecological risk assessment (*Equation 8*).^64^

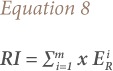
When the ecological risk (ER) is <40 and RI<150, this implies low ecological risk, while 40≤ER<80 and 150≤RI<300 indicate moderate ecological risk. A 80≤ER<160 and 300≤RI≤600 indicates considerable ecological risk, while 160≤ER<320 implies high ecological risk. An ER≥320 and RI>600 indicate very high ecological risk.

## Results

**Figure 2:**
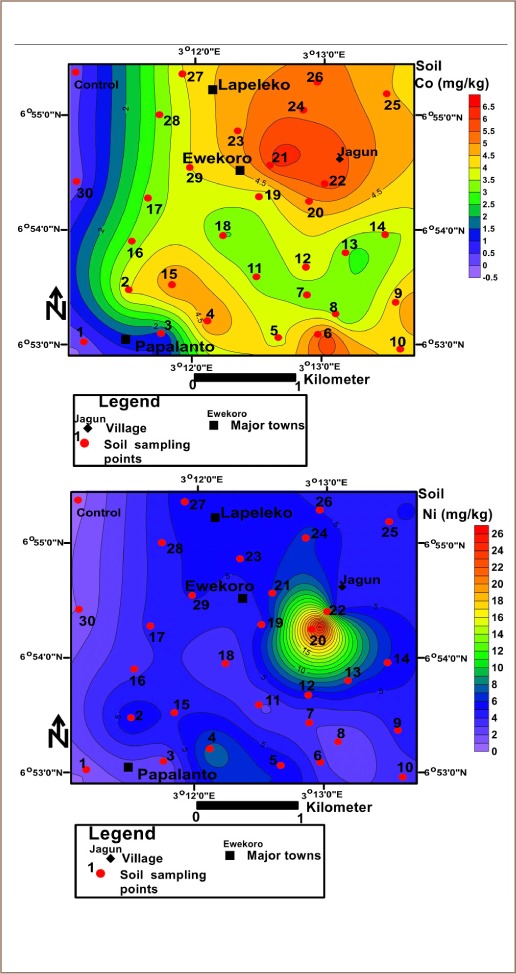
Spatial distribution of cobalt and nickel in topsoil in the study area

**Figure 3:**
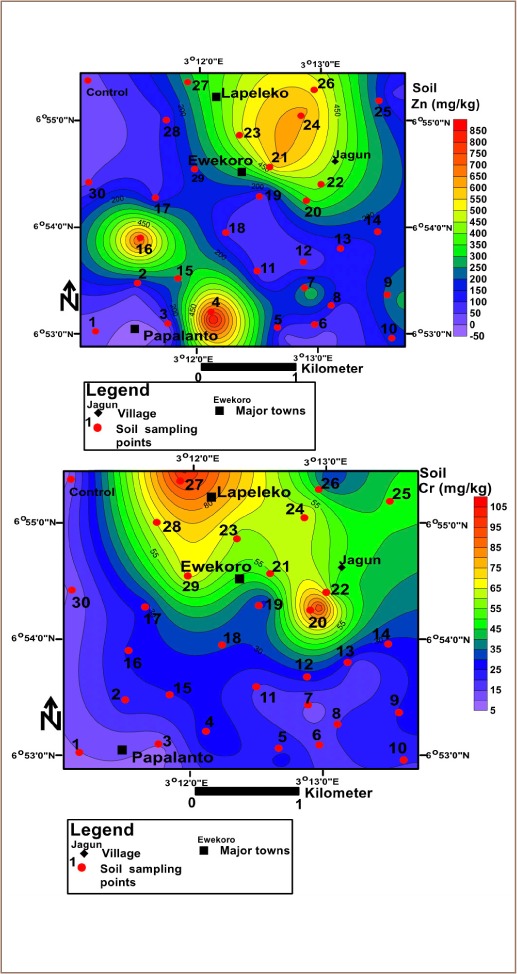
Spatial distribution of zinc and chromium in topsoil in the study area

**Figure 4:**
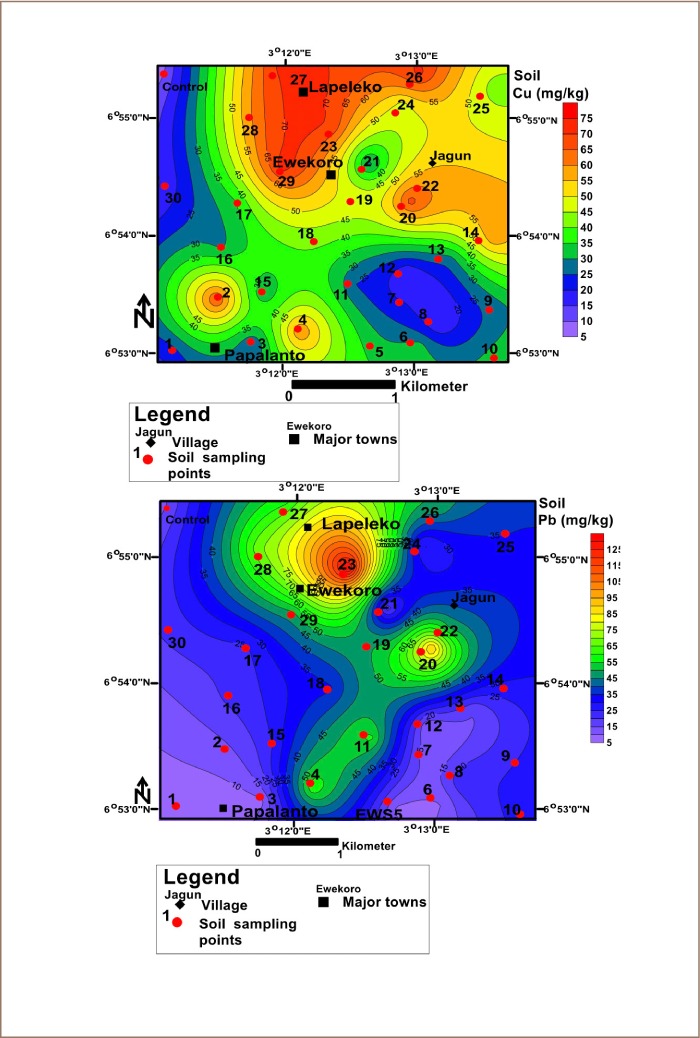
Spatial distribution of copper and lead in topsoil in the study area

The average concentrations of heavy metals in soils in the study area are presented in Table 1. The mean soil concentrations of the metals were Cu: 41.63 mg/kg; Pb: 35.43 mg/kg; Zn: 213.64 mg/kg; Cr: 35.60 mg/kg; Co: 3.84 mg/kg and Ni: 5.13 mg/kg *(Table 1).* The results revealed a decreasing order of Zn>Cu>Cr>Pb>Ni>Co *(Table 1)* for the metals concentrations. Sample 21 had the highest concentration of Co, while sample 30 has the least concentration (*Figure 2*). For Ni, sample 20 had the highest concentration, while sample 30 had the lowest (*Figure 2*). The concentration of Zn was highest in sample 4 and lowest in sample 1 *(Figure 3).* The concentration of Cr was highest in sample 27 and lowest in sample 1 *(Figure 3).* The highest concentration of Cu was found in sample 27, while the lowest was found in sample 1 *(Figure 4).* The highest concentration of Pb was found around sample 23, while the least values were found in sample 1 *(Figure 4).* The study further showed that the concentrations of Cr in soils in the study area were above the United States Environmental Protection Agency (USEPA) standards, while the concentrations of Zn, Cu, Pb, Ni and Co were below this standard.^65^ The average concentrations of the metals in soils were greater than those in the control samples (*Table 1*).

**Table 1:** Average Concentration of Heavy Metals in Soils and Plants of the Study Area

**Metal**	**Media**	**Mean (mg/kg)**	**Standard Deviation (mg/kg)**	**Minimum (mg/kg)**	**Maximum (mg/kg)**	**Significance value**	**Background value (mg/kg)**	**USEPA (2002)^65^ (mg/kg)**	**FAO (2001)^61^ (mg/kg)**	**Ashaka Cement (Wufem *et al*.,)^66^ (mg/kg)**	**Objana (Odoh *et al*.,)^67^ (mg/kg)**
**Cu**	**Soil**	41.63	20.01	15.25	72.18	ρ = 0.01	5.29	270		0.30	0.083
	**Plants**	26.52	52.81	2.59	305.6	ρ = 0.01			0.60		
**Pb**	**Soil**	35.43	27.92	8.00	132.45	ρ = 0.01	11.26	200		-	0.046
	**Plants**	15.46	28.51	0.66	109	ρ = 0.01			0.20		
**Zn**	**Soil**	213.64	231.71	14.00	877	ρ = 0.01	26.37	1100		9.80	0.262
	**Plants**	135.87	444.58	14.90	252	ρ = 0.01			0.60		
**Cr**	**Soil**	35.60	24.86	8.00	102.15	ρ = 0.01	5.91	11			
	**Plants**	30.62	63.62	1.20	343	ρ = 0.01			1.30		
**Co**	**Soil**	3.84	1.63	0.05	6.28	ρ = 0.01	0.08	-		27.37	0.026
	**Plants**	0.45	0.9	0.02	4.54	ρ = 0.01			-		
**Ni**	**Soil**	5.13	4.35	1.00	26.31	ρ = 0.01	0.22	72		2.56	0.035
	**Plants**	3.77	7.22	0.06	29.10	ρ = 0.01			0.11		

Abbreviations: USEPA, United States Environmental Protection Agency; FAO, Food and Agricultural Organization.

### Metal concentrations in plants

The decreasing order of metals in plants in the present study area are Zn>Cr>Cu>Pb>Ni>Co (*Table 1*). Zinc had a mean value of 135.87 mg/kg, a minimum value of 14.90 mg/kg and a maximum value of 252.00 mg/kg; with a significance of ρ = 0.01. Chromium had a mean value of 30.62 mg/kg, a minimum value of 1.20 mg/kg and a maximum of 343.00 mg/kg, with a significant value of ρ = 0.01. Copper had a mean value of 26.52 mg/kg, with a minimum value of 2.59 mg/kg and maximum value of 305.60 mg/kg. The concentrations of Zn, Cr, Cu, Pb, Ni and Co in plants are above CODEX recommended limits.^61^

### Contamination assessment of soils and plants

Results of the contamination assessment of heavy metals in soils in the study area are presented in Table 2. Copper had minimum and maximum Igeo values of 0.94 and 3.19, respectively, with an average of 2.23, while Pb had minimum and maximum Igeo values of −1.08 and 2.97, respectively, with a mean value of 0.69. Zinc showed a mean Igeo value of 1.84, while the average value of Igeo for Cr was 1.70. The minimum Igeo value for Co was −1.26, with a maximum value of 5.71 and a mean of 4.66, while the minimum Igeo value for Ni was 1.57 with a maximum Igeo value of 6.32. In addition, the results showed that the CF for Cu in soils of the area showed a minimum and maximum of 2.88 and 13.64, respectively, while for Pb the CF ranged between 0.71 and 11.76. The average CF value for Zn was 9.05 and 6.03 for Cr. For Co, the CF ranged between 0.63 and 78.58, and for Ni the minimum CF was 4.45 and the maximum was 119.59. The minimum CD for heavy metals in soils in the study area was 11.90, while the maximum was 105.82. In plants in the area, the mean contamination load index for Cu, Pb, Zn, Cr and Ni was 44.20, 77.30, 226.45, 23.55 and 34.27, respectively (*Table 3*).

**Table 2:** Geo-Accumulation Index, Contamination Factor, Contamination Degree and Pollution Load Index of Heavy Metals in Soils

**Metals**	**Igeo**			CF/CD*/PLI**		

	**Minimum**	**Maximum**	**Average**	**Minimum**	**Maximum**	**Average**
**Cu**	0.94	3.19	2.23	2.88	13.64	7.87
**Pb**	−1.08	2.97	0.69	0.71	11.76	3.15
**Zn**	−1.49	4.47	1.84	0.53	33.26	9.05
**Cr**	−0.15	3.53	1.70	1.35	17.28	6.03
**Co**	−1.26	5.71	4.66	0.63	78.58	47.99
**Ni**	1.57	6.32	3.68	4.45	119.59	23.33
				11.90*	105.82*	97.42*
				1 37**	23.76**	9.73**

**Table 3:** Bioaccumulation Factor and Contamination Load Index of Heavy Metals in Plants

**Metals**	**Bioaccumulation factor**			**Contamination load index**

	**Minimum**	**Maximum**	**Average**	**Average**
**Cu**	0.04	1.84	0.57	44.20
**Pb**	0.01	5.09	0.54	77.30
**Zn**	0.03	6.93	1.39	226.45
**Cr**	0.02	5.11	0.86	23.55
**Co**	0.01	7.40	0.43	-
**Ni**	0.05	3.87	0.78	34.27

### Bivariate correlation

The results of the significant values (> 0.01) *(Table 3)* revealed that all metals in plants and soils originated from the same source, with the exception of Zn, which showed a varied value in soils and plants, suggesting different sources for the two media. This was confirmed in the results of the bivariate correlation *(Table 4)* that revealed the following significant correlations: Pb-Cu (r=0.680); Cu-Cr (r=0.699); Co-Cu (r=0.553); Pb-Cr (r=0.638); Co-Zn (r=0.529); Co-Cr (r=0.509) and Co-Ni (r=0.624) in soil and (Cu: r=0.682; Pb: r=0.606; Zn: r=0.923; Cr: r=0.973 and Ni: r=0.924) in plants, implying that the sources of these metals in plants in the study area originate predominantly from uptake from soils.

**Table 4:** Bivariate Correlation of Heavy Metals in Soils and Plants

	**Cu_Plant_**	**Pb_Plant_**	**Zn_Plant_**	**Cr_Plant_**	**Co_Plant_**	**Ni_Plant_**	**Cu_Soil_**	**Pb_Soil_**	**Zn_Soil_**	**Cr_Soil_**	**Co_Soil_**	**Ni_Soil_**
**Cu_Plant_**	1											
**Pb_Plant_**	0.670^**^	1										
**Zn_Plant_**	0.966^**^	0.663^**^	1									
**Cr_Plant_**	0.930^**^	0.723^**^	0.895^**^	1								
**Co_Plant_**	0.872^**^	0.737^**^	0.855^**^	0.877^**^	1							
**Ni_Plant_**	0.926^**^	0.783^**^	0.911^**^	0.981^**^	0.933^**^	1						
**Cu_Soil_**	**0.682**	**0.752**	**0.862**	**0.746**	**0.927**	**0.817**	1					
**Pb_Soil_**	**0.666**	**0.606**	**0.546**	**0.522**	**0.995**	**0.517**	0.680^**^	1				
**Zn_Soil_**	**0.610**	0.240	**0.923**	**0.516**	**0.951**	**0.863**	0.390^**^	0.375^*^	1			
**Cr_Soil_**	**0.951**	0.226	**0.867**	**0.973**	**0.849**	**0.851**	0.699^**^	0.638^**^	0.379^*^	1		
**Co_Soil_**	**0.969**	0.247	**0.829**	**0.873**	0.376	**0.631**	0.553^**^	0.363^*^	0.529^**^	0.509^**^	1	
**Ni_Soil_**	**0.931**	**0.765**	**0.976**	**0.976**	**0.941**	**0.924**	0.473^**^	0.470^**^	**0.313**	0.624^**^	0.438^*^	1

^*^Correlation significant at 0.01 level

^**^ Correlation significant at 0.05 level

### Bioaccumulation assessment of heavy metals in plants

The outcomes of the bioaccumulation assessment of heavy metals in plants in the study area are shown in Table 3. The minimum and maximum bioaccumulation factor for Cu was 0.04 and 1.84, respectively, and ranged between 0.01 and 5.09 for Pb. The average bioaccumulation factor for Zn, Cr, Co and Ni was 1.39, 0.86, 0.43 and 0.78, respectively.

### Ecological risk

**Figure 5:**
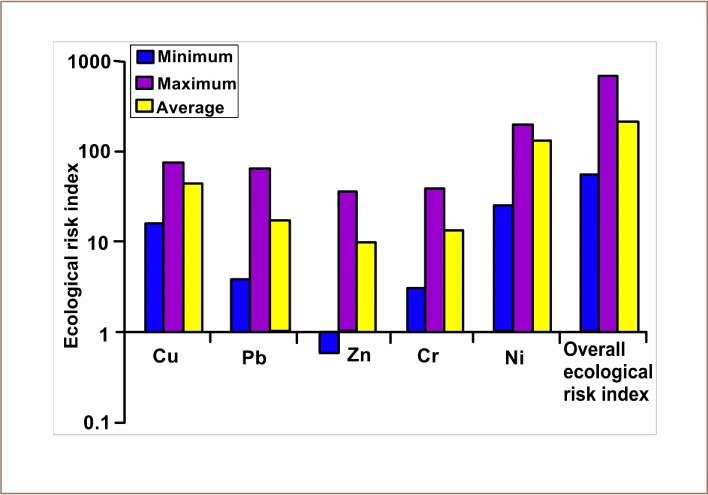
Ecological risk index of heavy metals in soils in the study area

Results of the ecological risk assessment of heavy metals in soils in the study area are presented in Figure 5. The minimum ERI for Cu was 14.41, while the maximum was 68.22. For Pb, the minimum and maximum ERI was 3.55 and 58.81, respectively. For Zn, the ERI ranged between 0.53 and 33.26, with an average of 9.05, while the minimum and the maximum ERI for Cr was 2.71 and 34.57, respectively. Nickel had a minimum and maximum ERI of 22.27 and 597.95, respectively. The overall ERI for all the metals ranged between 49.71 and 749, with an average of 350.26

## Discussion

Except for Cr in soils of Ewekoro, concentrations of heavy metals were below the recommended limits set by the USEPA.^65^ However, they were above their corresponding measured concentrations in the background soils *(Table 1).* Relating the above results with similar studies done in Ashaka and Obajana cement production areas in Nigeria, the concentrations of Cu, Pb, Zn and Ni in soils of the study area were above those reported in the two areas.^55–58^ However, concentrations of Co in soils were lower than those reported in Ashakasoils, and higher than those in Obajana soils. In addition, concentrations of potentially toxic metals in plants in this area were above the recommended limits.^60^ The spatial distribution of Co and Ni in soil *(Figure 2)* revealed that Jagun had higher concentrations of Co than other parts of the study area. In addition, Zn was highly concentrated in the northeast and southwest of the study area *(Figure 3).* Concentrations of Cr were high in the northwest around Ewekoro, Lapeleko and Jagun *(Figure 3).* Copper was well distributed in soils across the study area, but was highly concentrated around Ewekoro, Lapeleko, Jagun and Papalanto (*Figure 4*). Concentrations of Pb were high in soils around Ewekoro and Lapeleko *(Figure 4).* The study revealed that aerial deposition of metal-laden soils might have contributed significantly to their concentration in the area. A study by Afolabi *et al.* showed that more than 70% of the inhabitants of the area live in houses about 2 km from the cement processing factory.^45^ This indicates that toxic metals in soils of the area are highly concentrated in areas where people live. In addition, concentrations of heavy metals in plants of the area were above the recommended limits set by CODEX.^61^

In the study area, the Igeo revealed that soils are unpolluted to strongly polluted by Cu Pb and Cr (the classification has been clearly defined in Equation 1), while they are unpolluted to extremely polluted by Zn and Co *(Table 2)*. In addition, soils are moderately to extremely polluted by Ni (Loska *et al*).^56^ However, the results of the CF showed that the soils in the area ranged from lowly to very highly contaminated by Pb, Zn and Co, while Cu and Cr present moderate to very high contamination (the classification is defined in Equation 2). Nickel showed considerable to very high contamination in soils of this area. The CD showed that heavy metals in soils of the area pose a moderate to high degree of contamination *(Table 2).* This was affirmed by the PLI which also revealed a moderate to high degree of pollution *(Table 2).* This showed that cement production, processing, and transportation coupled with the abandoned railway track in the area might have significantly contributed to the high degree of contamination recorded in the area. According to Afolabi *et al.*, land and aerial pollution contributed 6.40% and 80.81% of the total pollution in the study area.^45^ Oral ingestion, dermal contact and inhalation of contaminated soils and dusts might have contributed significantly to the spread of diseases in the area as reported by Afolabi *et al.*^45^ In addition, the contamination load index revealed that plants in this area are highly contaminated by heavy metals. Consumption of contaminated vegetables and plants may also contribute to health issues in the study area.

The present study further revealed the presence of metals transfer from soil to plants across most of the study area *(Table 3).* Bivariate correlation *(Table 4)* revealed that Cu, Pb, Zn, Cr and Ni were mobilized from soils to plants, while Co was not. Although metals in plants might have originated from soils in the area, aerial deposition of contaminated dusts is another possible means of contamination, entering plants through their stomata.

Correlation analysis of metals in soils showed that the potentially toxic elements in soils of the area might have originated from common mixed anthropogenic and point sources. Major possible sources of metals in soils of the area are cement production, processing, processing, rail and vehicular transportation. It was observed that Cu, Pb, Zn and Cr in soils of the study area posed low to considerable ecological risk (*Figure 5*), while Ni posed considerable to very high ecological risk (the classification is defined in Equation 8).

## Conclusions

The present study was carried out to assess the extent of heavy metals contamination and their potential ecological risk in soils and plants of Ewekoro, southwest Nigeria. Concentrations of heavy metals in soils and plants in the area were above those in background samples and cement production areas across the country. Soils and plants in the area are contaminated by heavy metals which possibly originate from anthropogenic activities, especially from cement production and processing as well as rail and vehicular transportation. In addition, crops in the area are strong bioaccumulators of these heavy metals, although aerial deposition of contaminated dust is also a potential source of metals. Furthermore, the ecological risk potential of heavy metals in soils of the area ranged from low to considerably high. Further studies should be conducted on the extent of heavy metal bioaccumulation in this area and the potential health risk to local residents. Stricter rules should be introduced to regulate cement production activities to lower the emission rate of cement dust polluted with metals into the environment.
